# Opportunistic assessment of steatotic liver disease in lung cancer screening eligible individuals

**DOI:** 10.1111/joim.20053

**Published:** 2025-01-27

**Authors:** Jakob Weiss, Simon Bernatz, Justin Johnson, Vamsi Thiriveedhi, Raymond H. Mak, Andriy Fedorov, Michael T. Lu, Hugo J. W. L. Aerts

**Affiliations:** ^1^ Artificial Intelligence in Medicine (AIM) Program Mass General Brigham Harvard Medical School Harvard Institutes of Medicine (HIM) Boston Massachusetts USA; ^2^ Department of Radiation Oncology Brigham and Women's Hospital Dana‐Farber Cancer Institute, Harvard Medical School Boston Massachusetts USA; ^3^ Department of Radiology Brigham and Women's Hospital Dana‐Farber Cancer Institute Harvard Medical School Boston Massachusetts USA; ^4^ Department of Diagnostic and Interventional Radiology Faculty of Medicine University Medical Center Freiburg University of Freiburg Freiburg Germany; ^5^ Radiology and Nuclear Medicine CARIM & GROW Maastricht University Maastricht The Netherlands; ^6^ Cardiovascular Imaging Research Center Massachusetts General Hospital Harvard Medical School Boston Massachusetts USA

**Keywords:** artificial intelligence, CT imaging, lung cancer screening, opportunistic, risk assessment, steatotic liver disease

## Abstract

**Background:**

Steatotic liver disease (SLD) is a potentially reversible condition but often goes unnoticed with the risk for end‐stage liver disease.

**Purpose:**

To opportunistically estimate SLD on lung screening chest computed tomography (CT) and investigate its prognostic value in heavy smokers participating in the National Lung Screening Trial (NLST).

**Material and methods:**

We used a deep learning model to segment the liver on non‐contrast‐enhanced chest CT scans of 19,774 NLST participants (age 61.4 ± 5.0 years; 41.2% female) at baseline and on the 1‐year follow‐up scan if no cancer was detected. SLD was defined as hepatic fat fraction (HFF) ≥5% derived from Hounsfield unit measures of the segmented liver. Participants with SLD were categorized as lean (body mass index [BMI] < 25 kg/m^2^) and overweight (BMI ≥ 25 kg/m^2^). The primary outcome was all‐cause mortality. Cox proportional hazard regression assessed the association between (1) SLD and mortality at baseline and (2) the association between a change in HFF and mortality within 1 year.

**Results:**

There were 5.1% (1000/19,760) all‐cause deaths over a median follow‐up of 6 (range, 0.8–6) years. At baseline, SLD was associated with increased mortality in lean but not in overweight/obese participants as compared to participants without SLD (hazard ratio [HR] adjusted for risk factors: 1.93 [95% confidence interval 1.52–2.45]; *p* = 0.001). Individuals with an increase in HFF within 1 year had a significantly worse outcome than participants with stable HFF (HR adjusted for risk factors: 1.29 [1.01–1.65]; *p* = 0.04).

**Conclusion:**

SLD is an independent predictor for long‐term mortality in heavy smokers beyond known clinical risk factors.

## Graphical abstract

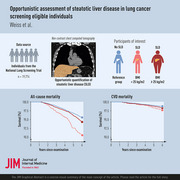

**Abbreviations**
BMI: body mass index
CI: confidence interval
CT: computed tomography
HR: hazard ratio
HUs: Hounsfield units
ICD: Internationals Statistical Classification of Diseases
IQR: interquartile range
NLST: National Lung Screening Trial
SLD: steatotic liver disease

## Introduction

Steatotic liver disease (SLD) is a leading cause of end‐stage liver disease and cancer and has also been identified as an independent risk factor for cardiovascular disease and major adverse cardiovascular events [1, 2]. With an estimated prevalence of up to 30% in the United States [3, 4], SLD is of major socioeconomic importance, emphasizing the urgent need for early diagnosis to reduce morbidity and mortality [5]. However, as SLD is usually a subclinical and asymptomatic condition, affected individuals are often unaware of their increased risk, and actionable, preventive measures such as lifestyle inventions, diet, and exercise are not initiated. This is particularly true for lean individuals (body mass index [BMI] < 25 kg/m^2^) with SLD, who are increasingly recognized as a distinct population with a specific pathophysiological profile that is associated with a higher risk for mortality compared to overweight and obese subjects with SLD [3, 6, 7, 8] highlighting the need for novel strategies for early and reliable identification.

Liver biopsy is currently the reference standard for diagnosing SLD but has an associated risk of procedural complications, sampling error, and high cost. Besides magnetic resonance imaging and ultrasound, non‐contrast‐enhanced computed tomography (CT) has been suggested as a noninvasive alternative for diagnosis based on changes in hepatic tissue characteristics due to accumulating fat and has demonstrated a strong correlation with biopsy‐obtained fat content [9, 10, 11]. Thus, CT‐based quantification of SLD may serve as a reproducible and accurate method to identify individuals at risk [12, 13], which might be particularly relevant in populations that undergo CT imaging in a regular and organized fashion, such as lung cancer screening eligible individuals. With the recent introduction of the 2021 US Preventive Services Task Force Recommendation on Lung Cancer Screening [14], which broadened the eligibility criteria, an estimate of 15 million Americans qualify for screening. This is almost twice as high compared to the previous recommendations [15] and holds enormous potential for opportunistic screening of SLD, which has now become feasible in an automated fashion with novel artificial intelligence techniques [16, 17, 18].

In this study, we used a fully automated deep learning model to estimate SLD from lung screening chest CTs of the National Lung Screening Trial (NLST). NLST was a randomized controlled trial that enrolled heavy smokers for lung cancer screening via chest CT versus chest radiographs [19]. Participants were imaged at baseline and received up to two annual follow‐up scans. Our study investigates the prognostic value of opportunistic SLD quantification to predict mortality using the baseline and 1‐year change in hepatic fat fraction (HFF). We found a strong association between SLD and mortality, in particular in lean participants. In addition, an increase in HFF within 1 year was strongly associated with an increased risk of mortality independent of baseline SLD. Our results demonstrate that automated methods can provide a fast, low‐cost, and noninvasive solution to identify individuals with SLD and hold the potential to accelerate clinical implementation to guide preventive measures and improve population health.

## Methods

**Fig. 1:**
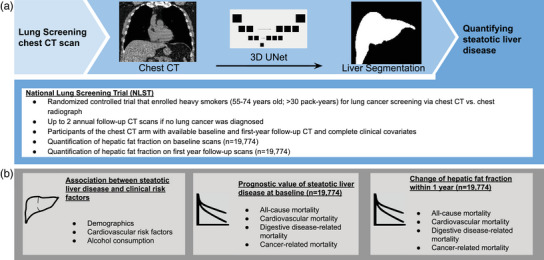
**Overview of the study design**. (a) We used a fully automated deep learning model to quantify steatotic liver disease on lung cancer screening chest computed tomography (CT) from participants of the National Lung Screening Trail. (b) Next, we investigated the association of steatotic liver disease with clinical risk factors and explored the prognostic value of steatotic liver disease to predict mortality at baseline as well as the change in hepatic fat fraction between the baseline and first‐year follow‐up CT. BMI, body mass index; NLST, National Lung Screening Trial.

An overview of the study design is provided in Fig. 1.

### Study population

**Fig. 2:**
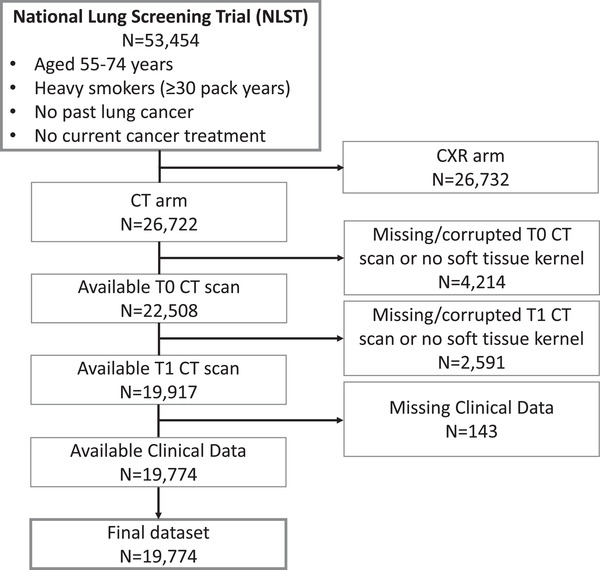
Consort diagram.

Participants for this study were drawn from NLST—a randomized controlled multicenter trial for lung cancer screening via chest CT versus chest radiograph [19]. All participants were heavy smokers (>30 pack years) aged 55–74 years who received a baseline scan at enrollment and up to two annual follow‐up scans if they were not diagnosed with lung cancer. A total of 53,454 participants were enrolled. Among those, 26,722 randomly selected individuals underwent low‐dose CT imaging. Details on the image acquisition and reconstruction parameters are provided in the Supporting Information. For analysis, only participants with available baseline and first‐year follow‐up scan and a predefined set of clinical covariates were included. The following covariates were considered: age in years, BMI (kg/m^2^), sex, race (White, African American, others), smoking status (former vs. current), pack‐years, prevalent hypertension, prevalent type II diabetes, past myocardial infarction, stroke, and alcohol consumption (at least four alcoholic beverages per week). A total of 6805 participants were excluded due to corrupted imaging data or because no baseline and/or first‐year follow‐up scan was available. Additional 143 participants were excluded due to missing clinical covariates resulting in a final study cohort of 19,774 individuals (Fig. 2).

All NLST participants provided written informed consent. Secondary use was approved by the National Cancer Institute, Bethesda, Maryland, and Partners Healthcare, Boston, MA, institutional review board.

### Deep learning model for automatic quantification of steatotic liver disease

We used the open‐source TotalSegmentator [20] tool for fully automated volumetric segmentation of the part of the liver covered in the lung screening chest CT scan, which was on average around two thirds of the entire organ. *Pyradiomics* [21] open‐source library was used to extract mean liver density in Hounsfield units (HUs) within the segmentation mask. Computation of the segmentation and density extraction was implemented on the Broad Terra platform as previously described [22], with the analysis results publicly available [23].

### Definition of steatotic liver disease and subgroups

Traditionally, SLD is defined as a mean hepatic attenuation <40 HUs [11, 13, 24], which is evaluated manually by placing one or more region(s)‐of‐interest in the liver and intentionally sparing large vessels [25]. This approach has been shown to be strongly correlated with biopsy‐obtained fat content (>30%) and validated as a reproducible and reliable measure to define SLD on non‐contrast‐enhanced CT imaging [11, 12]. More recently, Graff et al. [26] suggested an approach to convert the raw CT HU values into an HFF equivalent to the proton‐density fat fraction known from magnetic resonance imaging. This approach was also used in the present study by calculating the HFF based on the mean HU values in the deep learning‐generated volumetric liver segmentation mask as follows:

$$\mathrm{Hepatic}\;\mathrm{fat}\;\mathrm{fraction}={\mathrm{CT}}_{\mathrm{HU}}\times \left(-0.58\right)+38.2$$

After conversion of the HU values into an HFF, SLD was defined as an HFF of ≥5% as this threshold is known to indicate mild steatosis. For CT HU values resulting in an apparently negative HFF (HU ≥ 65.9), the fat fraction was set to 0%. Further, the HFF was categorized into three groups using established thresholds (5%–14% = mild SLD; >14%–28% = moderate SLD; >28% = severe SLD) [11]. Additionally, patients were stratified by their (BMI < 25 vs. ≥25 kg/m^2^) to identify and compare lean versus overweight/obese effects of SLD [6].

### Clinical endpoints

The primary endpoint of this study was all‐cause mortality. Secondary endpoints were death caused by cardiovascular disease, cancer, and digestive disease based on the International Statistical Classification of Diseases 10th revision (ICD‐10) codes “I*,” “C*,” or “K*,” respectively. Follow‐up data were truncated at 6 years. Death was assessed via annual questionnaires and linkage to the National Death Index as reported in the parent trial.

### Statistical analysis

All statistical analyses were performed in R (version 4.2.2). Continuous variables are given as mean or median with standard deviation or interquartile range; categorical variables as frequencies and percentages.

Time‐to‐event analyses used Kaplan–Meier survival estimates and univariable and multivariable Cox proportional hazard regression analysis adjusted for the abovementioned clinical covariates. First, participants at baseline were stratified into three groups: (1) reference group with no SLD, (2) overweight/obese participants (BMI ≥ 25 kg/m^2^) with SLD, and (3) lean participants (BMI < 25 kg/m^2^) with SLD to explore the association of SLD and mortality. In addition, the relative change in the HFF, defined as a ≥5% point increase from baseline to the first‐year follow‐up scan adjusted for baseline SLD, was calculated and the association with mortality investigated. A *p*‐value < 0.05 was considered to indicate statistical significance.

## Results

### Study population

A total of 19,774 heavy smoking individuals of the NLST, screened with non‐enhanced chest CT for lung cancer, were included in this study. Of these, 11,934 (60%) did not have SLD, 6787 (34%) were overweight or obese (BMI ≥ 25 kg/m^2^) with SLD, and 1053 (5%) were lean (BMI < 25 kg/m^2^) with SLD. Of the total cohort, 11,627 (59%) were men, the mean age was 61.4 ± 5 years, and the majority of the participants were White 18,534 (94%). The median follow‐up was 6 ± 0.6 years for all participants. Further detailed demographics are provided in Table 1a.

**Table 1a:** Baseline demographics.

Characteristic	Entire cohort	No SLD	SLD, BMI ≥ 25	SLD, BMI < 25
**Participants, *n***	19,774	11,934	6787	1053
**SLD at baseline**	39.6% (7840/19,774)	0% (0/11,934)	100% (6787/6787)	100% (1053/1053)
**Mean age, years, mean (SD)**	61.4 ± 5	61.3 ± 5	61.4 ± 4.9	62.3 ± 5.2
**Sex**				
Female	41.2% (8147/19,774)	47.6% (5678/11,934)	30% (2034/6787)	41.3% (435/1053)
Male	58.8% (11627/19774)	52.4% (6256/11934)	70% (4753/6787)	58.7% (618/1053)
**Race**				
White	93.7% (18,534/19,774)	93.5% (11,153/11,934)	94.2% (6396/6787)	93.5% (985/1053)
Black	4.2% (834/19,774)	4.8% (577/11,934)	3.2% (218/6787)	3.7% (39/1053)
Other	1.7% (334/19,774)	1.5% (174/11,934)	2% (134/6787)	2.5% (26/1053)
Unknown	0.4% (72/19,774)	0.3% (30/11,934)	0.6% (39/6787)	0.3% (3/1053)
**BMI**				
Underweight	0.8% (153/19,774)	1.1% (132/11,934)	0% (0/6787)	2% (21/1053)
Normal	27.7% (5486/19,774)	37.3% (4454/11,934)	0% (0/6787)	98% (1032/1053)
Overweight	42.6% (8430/19,774)	43.3% (5168/11,934)	48.1% (3262/6787)	0% (0/1053)
Obese	28.9% (5705/19,774)	18.3% (2180/11,934)	51.9% (3525/6787)	0% (0/1053)
**Diabetes**	9.4% (1856/19,774)	5.6% (672/11,934)	16.2% (1100/6787)	8% (84/1053)
**Smoking status**				
Former smoker	52.8% (10,441/19,774)	48.6% (5805/11,934)	61.9% (4203/6787)	41.1% (433/1053)
Current smoker	47.2% (9333/19,774)	51.4% (6129/11,934)	38.1% (2584/6787)	58.9% (620/1053)
**Pack years, mean (SD)**	55.7 ± 23.6	54.1 ± 22.3	58.2 ± 25.2	57.5 ± 24.3
**Alcohol, ≥4/W**	29.1% (5101/17,557)	26.7% (2791/10,450)	30.4% (1870/6153)	46.1% (440/954)
**Past myocardial infarction**	13% (2564/19,774)	11.4% (1364/11,934)	16% (1086/6787)	10.8% (114/1053)
**Past stroke**	2.8% (546/19,774)	2.7% (328/11,934)	2.8% (193/6787)	2.4% (25/1053)
**Hypertension**	34.8% (6882/19,774)	29.1% (3470/11,934)	45.1% (3061/6787)	33.3% (351/1053)
**All‐cause mortality events**	5.1% (1000/19,760)	4.4% (519/11,923)	5.6% (383/6786)	9.3% (98/1051)
**CVD mortality events**	1.4% (285/19,760)	1.2% (149/11,923)	1.7% (113/6786)	2.2% (23/1051)
**Cancer mortality events**	1.8% (358/19,760)	1.6% (188/11,923)	2% (135/6786)	3.3% (35/1051)
**Digestive disease mortality events**	0.2% (30/19,760)	0.1% (12/11,923)	0.2% (12/6786)	0.6% (6/1051)
**Follow‐up, years, median (SD)**	6 (0.6)	6 (0.6)	6 (0.6)	6 (0.6)

*Note*: Missingness for alcohol consumption was 2217 and for cause‐specific death was 14. Missingness for alcohol consumption in patients with known cause of death was 2210, as stated in adjusted survival analyses.

Abbreviations: BMI, body mass index; IQR, interquartile range; SD, standard deviation; SLD, steatotic liver disease; W, weeks.

### Association between steatotic liver disease and mortality

**Fig. 3:**
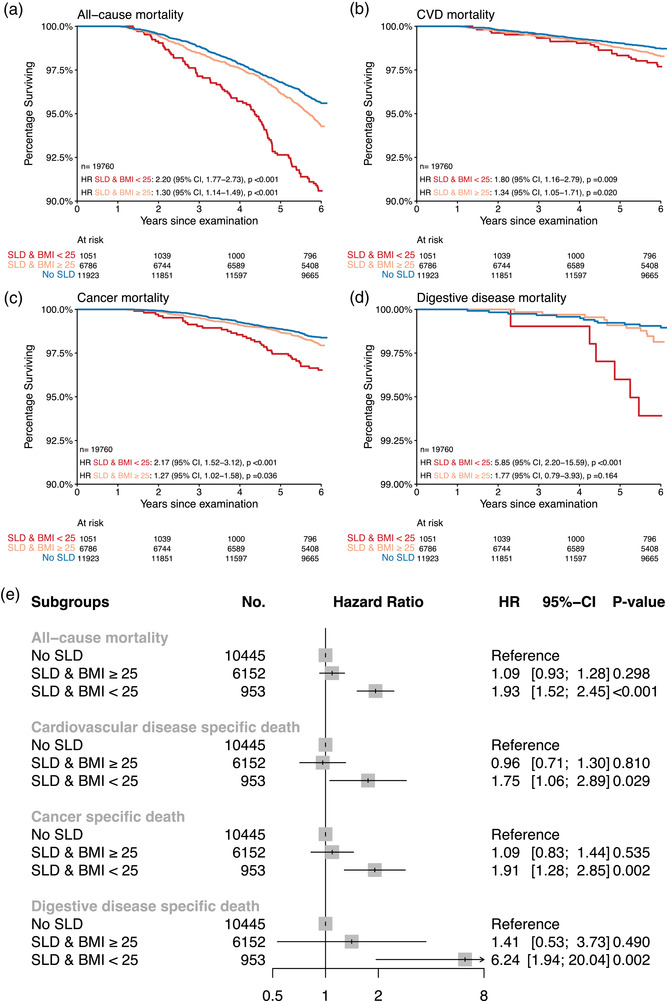
**Association between steatotic liver disease and mortality at baseline**. Kaplan–Meier survival curves for steatotic liver disease by body mass index (BMI) groups for (a) all‐cause, (b) cardiovascular disease (CVD), (c) cancer, and (d) digestive disease mortality. The inserts show univariate Cox regression analyses versus the reference of no steatotic liver disease. (e) Forest plots with multivariable‐adjusted hazard ratios (HR) and 95% confidence intervals (CI), including age, BMI, sex, race, smoking status, pack‐years, prevalent hypertension, prevalent type II diabetes, past myocardial infarction, stroke, and alcohol consumption as covariates. Unknown alcohol consumption in 2210 patients. HR, hazard ratio; SLD, steatotic liver disease.

First, we investigated the association between SLD and long‐term mortality. There were 5.1% (1000/19,760) deaths of all‐cause over a median follow‐up of 6.0 ± 0.6 years. Univariable Cox proportional hazard regression revealed that lean participants with SLD were more than twice as likely to die through 6 years of follow‐up as compared to participants without SLD (hazard ratio [HR], 2.20; 95% confidence interval [CI], 1.77–2.73; *p* < 0.001) (Fig. 3a). A similar but weaker association was found for overweight/obese individuals with SLD (HR, 1.30; 95% CI, 1.14–1.49; *p* < 0.001) as compared to participants without SLD. This signal remained robust after multivariable adjustment for age, BMI, sex, race, smoking status, pack‐years, prevalent hypertension, prevalent type II diabetes, past myocardial infarction, stroke, and alcohol consumption for lean but not for overweight/obese individuals (HR, 1.93; 95% CI, 1.52–2.45; *p* < 0.001). A subanalysis assessing the association between different SLD groups and all‐cause mortality and by female and male separately are provided in Fig. S1‐S3.

Similar associations were found for death due to cardiovascular disease, cancer, and digestive disease (Fig. 3b–e).

### Association between change in hepatic fat fraction and long‐term mortality

**Fig. 4:**
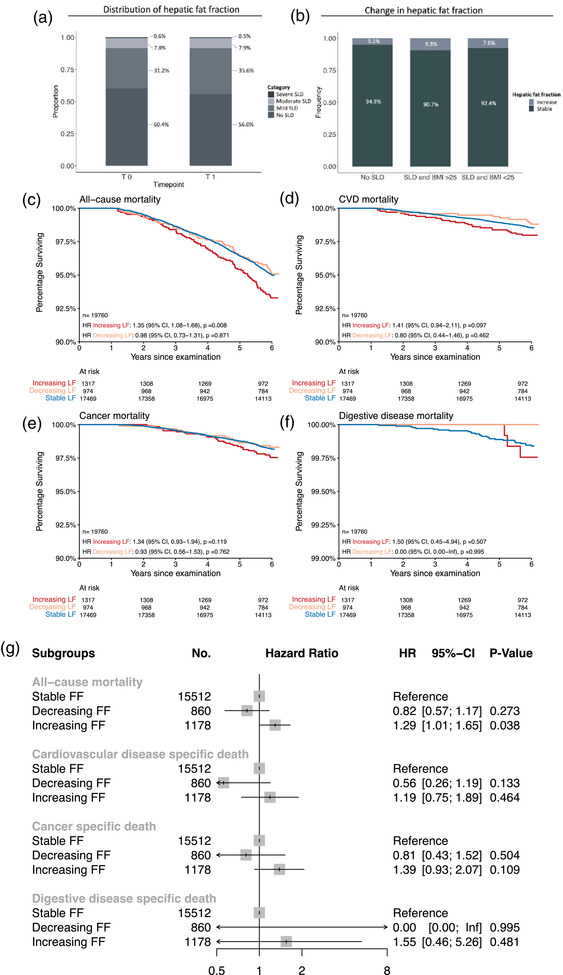
**Distribution and effect of change in hepatic fat fraction on long‐term mortality**. (a) Distribution of hepatic fat fraction at baseline (T0) and 1‐year follow‐up (T1) by hepatic fat fraction categories (0 = hepatic fat fraction < 5%, no SLD; 1 = hepatic fat fraction ≥ 5%–14%, mild SLD; 2 = hepatic fat fraction ≥ 15%–28%, moderate SLD; 3 = hepatic fat fraction > 28%, severe SLD). (b) Change in hepatic fat fraction (≥5% points) between T0 and T1 in individuals with no steatotic liver disease and individuals with steatotic liver disease stratified by BMI (≥25 vs. <25 kg/m^2^). Kaplan–Meier survival curves for change in hepatic fat fraction within 1 year from baseline for (c) all‐cause, (d) CVD, (e) cancer, and (f) digestive disease mortality. The inserts show univariate Cox regression analyses versus the reference of stable fat fraction.(g) Forest plots with multivariable‐adjusted hazard ratios (HRs) and 95% confidence intervals (CI) including baseline fat fraction, age, BMI, sex, race, smoking status, pack‐years, prevalent hypertension, prevalent type II diabetes, past myocardial infarction, stroke, and alcohol consumption as covariates. Unknown alcohol consumption in 2210 patients. BMI, body mass index; CVD, cardiovascular disease; FF, fat fraction; HR, hazard ratio; SLD, steatotic liver disease; T0, CT scan at baseline; T1, CT scan at 1‐year follow‐up.

Next, we investigated the change in the HFF between the baseline and first‐year follow‐up chest CT. Within 1‐year follow‐up, the prevalence of SLD increased by 4.4% from 39.6% at baseline to 44% at 1‐year follow‐up. This increase was observed to the vast majority in individuals with mild SLD (31.2% at baseline to 35.6% at follow‐up) (Fig. 4a and Fig. S2).

In the BMI‐stratified subgroups, the largest increase in SLD (9.3%) within 1 year was found in overweight/obese individuals, who already presented with SLD at baseline followed by lean individuals with SLD (7.6%) and individuals without SLD at baseline (5.1%) (Fig. 4b and Fig. S5).

At 1‐year follow‐up, a total of 1318 individuals had increased liver fat, 974 individuals had decreased liver fat, and 17,482 individuals had stable liver fat. Participants with increase in liver fat were slightly younger and more likely male as compared to individuals with stable liver fat at follow‐up. Participants with decrease in liver fat were more likely to be obese and consumed more alcoholic beverages at baseline as compared to individuals with stable liver fat (Table 1b).

**Table 1b:** Baseline demographics of participants with stable, decreasing, or increasing liver fat fraction.

Characteristic	Entire cohort	Stable liver ff	Decreasing liver ff	Increasing liver ff
**Participants, *n***	19,774	17,482	974	1318
**SLD at baseline**	39.6% (7840/19,774)	35.2% (6154/17,482)	100% (974/974)	54% (712/1318)
**Mean age, years, mean (SD)**	61.4 ± 5	61.5 ± 5	61.1 ± 4.7	60.8 ± 4.8
**Sex**				
Female	41.2% (8147/19,774)	41.6% (7270/17,482)	41.7% (406/974)	35.7% (471/1318)
Male	58.8% (11,627/19,774)	58.4% (10,212/17,482)	58.3% (568/974)	64.3% (847/1318)
**Race**				
White	93.7% (18,534/19,774)	93.8% (16,393/17,482)	93.4% (910/974)	93.4% (1231/1318)
Black	4.2% (834/19,774)	4.3% (755/17,482)	3.9% (38/974)	3.1% (41/1318)
Other	1.7% (334/19,774)	1.6% (275/17,482)	2% (19/974)	3% (40/1318)
Unknown	0.4% (72/19,774)	0.3% (59/17,482)	0.7% (7/974)	0.5% (6/1318)
**BMI**				
Underweight	0.8% (153/19,774)	0.8% (145/17,482)	0.3% (3/974)	0.4% (5/1318)
Normal	27.7% (5486/19,774)	29.5% (5160/17,482)	10% (97/974)	17.4% (229/1318)
Overweight	42.6% (8430/19,774)	43.1% (7531/17,482)	34.9% (340/974)	42.4% (559/1318)
Obese	28.9% (5705/19,774)	26.6% (4646/17,482)	54.8% (534/974)	39.8% (525/1318)
**Diabetes**	9.4% (1856/19,774)	8.7% (1527/17,482)	17% (166/974)	12.4% (163/1318)
**Smoking status**				
Former smoker	52.8% (10,441/19,774)	52% (9086/17,482)	65.1% (634/974)	54.7% (721/1318)
Current smoker	47.2% (9333/19,774)	48% (8396/17,482)	34.9% (340/974)	45.3% (597/1318)
**Alcohol, ≥4/W**	29.1% (5101/17,557)	28.8% (4475/15,518)	31.2% (268/860)	30.4% (358/1179)
**Pack years, mean (SD)**	55.7 ± 23.6	55.5 ± 23.4	58.5 ± 25.7	56.4 ± 23.6
**Past myocardial infarction**	13% (2564/19,774)	12.7% (2228/17,482)	14.9% (145/974)	14.5% (191/1318)
**Past stroke**	2.8% (546/19,774)	2.8% (489/17,482)	2.8% (27/974)	2.3% (30/1318)
**Hypertension**	34.8% (6882/19,774)	33.7% (5883/17,482)	47.1% (459/974)	41% (540/1318)
**All‐cause mortality events**	5.1% (1000/19,760)	5% (866/17,469)	4.8% (47/974)	6.6% (87/1317)
**CVD mortality events**	1.4% (285/19,760)	1.4% (248/17,469)	1.1% (11/974)	2% (26/1317)
**Cancer mortality events**	1.8% (358/19,760)	1.8% (311/17,469)	1.6% (16/974)	2.4% (31/1317)
**Digestive disease mortality events**	0.2% (30/19,760)	0.2% (27/17,469)	0% (0/974)	0.2% (3/1317)
**Follow‐up, years, median (SD)**	6 (0.6)	6 (0.6)	6 (0.6)	6 (0.6)

*Note*: Missingness for alcohol consumption was 2217 and for cause‐specific death was 14. Missingness for alcohol consumption in patients with known cause of death was 2210, as stated in adjusted survival analyses.

Abbreviations: BMI, body mass index; ff; fat fraction; IQR, interquartile range; SD, standard deviation; SLD, steatotic liver disease; W, weeks.

In Kaplan–Meier and Cox proportional hazard survival analysis, we found that participants with increasing HFF at 1‐year follow‐up were one third more likely to die through 6 years of follow‐up as compared to participants with stable HFF (HR, 1.35; 95% CI, 1.08–1.68; *p* = 0.008) (Fig. 4c). The increased risk of all‐cause death in participants with increasing liver fat at 1‐year follow‐up was preserved independent of baseline liver fat and further multivariable adjustment for age, BMI, sex, race, smoking status, pack‐years, prevalent hypertension, prevalent type II diabetes, past myocardial infarction, stroke, and alcohol consumption as covariates (HR, 1.29; 95% CI, 1.01–1.65; *p* = 0.04).

Similar trends were found across the disease‐specific causes of death, including cardiovascular disease, cancer, and digestive disease without reaching significance (Fig. 4d–g).

## Discussion

In this study, we demonstrate that fully automatic deep learning‐based opportunistic screening for SLD using lung screening chest CT can serve as an independent prognostic measure for long‐term mortality in lung cancer screening eligible individuals. We found that lean individuals with SLD were twice as likely to die through 6 years of follow‐up as compared to individuals without SLD and showed a more than sixfold increased risk to die from digestive diseases independent of known clinical risk factors. Similar results were found for cardiovascular and cancer‐related deaths. With an overall SLD prevalence of 39.6% and a 1‐year increase of approximately 4%—assuming a linear trend across the total lung cancer screening population of 15 million in the US (5.9 million and 600,000 individuals, respectively)—this opportunistic approach holds potential for fast, early, and actionable diagnosis of SLD beyond currently established strategies. This is of clinical importance as SLD is an underdiagnosed condition, and opportunistic, noninvasive identification of individuals at risk has the potential to improve personalized prevention and informed risk assessment to prevent progression to irreversible liver damage and mortality. Even when considering the relatively low screening adherence of 5.7% reported in the 2022 State of Lung Cancer report by the American Lung Association, automatic and opportunistic quantification of available but currently unused imaging information could help to act and improve population health.

Obesity is considered a key factor for the development of SLD [24, 25]. Yet, our results suggest that especially lean individuals with a BMI < 25 kg/m^2^ with SLD are at increased risk of detrimental outcomes as compared to overweight/obese individuals with SLD and individuals without SLD. Here, traditional metabolic risk assessment might fail to identify the individuals at highest risk, which could be addressed by the proposed opportunistic screening approach with little additional cost and without disruption of established workflows. Our results are in line with previous studies and suggest the existence of a lean SLD population with a different, yet poorly understood pathophysiology that is at greater risk for worse outcome and underdiagnosis due to the absence of typical risk factors [3, 6, 27]. Although the exact mechanism of pathogenesis remains still unclear, recent research has suggested links with epigenetic changes, genetic variants, and adaptive metabolic responses [28]. We extend these observations to a heavy smoking population that undergoes lung cancer screening CT on a regular basis and might not be diagnosed with SLD otherwise.

When focusing on a change in HFF over time, the prevalence of SLD increased by around 4% within 1‐year follow‐up with the largest increase in baseline overweight or obese individuals. Individuals with an increase in HFF had an elevated risk of death compared to those with stable HFF independent of cardiovascular risk factors and baseline HFF. Opportunistic assessment of SLD using longitudinally repeated screening examinations yields high potential to leverage changes over time to improve the risk profile of patients on a rolling basis.

Currently, chemical‐shift‐encoded MR imaging is considered the reference standard for noninvasive SLD assessment [10]. In addition, non‐contrast‐enhanced CT has been proposed as a value alternative given higher correlation with MRI results [9]. In this context, several deep learning approaches have been suggested to automatically quantify SLD from unenhanced chest or abdominal CT scans demonstrating the feasibility for population‐based opportunistic screening [26, 29, 30]. However, none of them investigated the association between SLD and patient outcome. The results of this study suggest that implementing a tool like the proposed deep learning model into the Picture Archiving and Communication System could allow for opportunistic and noninvasive diagnosis of SLD at high speed and low additional cost in examinations performed for various indications. Moreover, as no human input is needed, the implementation of such a tool could be done with minimal disruption of current clinical workflows and provide significant additional value to the clinician and patient by extracting relevant prognostic information to guide potential preventive or regenerative strategies.

There are limitations to our study. It is important to note that the participants of the study cohort were all heavy smokers participating in a lung cancer screening trial and mainly White. Additional studies are necessary to investigate whether these findings generalize to other, more diverse populations, which would broaden the applicability of the proposed approach. Second, several clinical risk factors were not available (e.g., liver enzymes) or self‐reported (such as alcohol consumption and pack years), which should be kept in mind when interpreting the results, in particular for lean individuals. Third, we investigated the association between SLD and digestive disease–related deaths as a composite endpoint, including liver disease–related deaths as only 12 individuals developed hepatocellular carcinoma and 16 individuals died from liver disease–related deaths, which does not allow for meaningful analysis. We therefore analyzed the impact of SLD on digestive disease–related deaths that include liver diseases together with other diseases of digestive organs such as the intestines or gallbladder. Fourth, the equation to calculate the HFF from HUs was developed for CT scans acquired at 120 kVp [9]. Alterations in tube voltage will cause small but measurable deviations, which we consider a minor but mentionable limitation, as 92% of all scans (35,464/38,548) included in this study were acquired at 120 kVp. Fifth, imaging was conducted using scanners of various vendors, types, and imaging protocols, which could be a potential source of confounding yet all scanners and imaging protocols were certified for use in the trial and protocol settings complied with the American College of Radiology guidelines to ensure a general level of comparability across imaging sites. Finally, integrating deep learning tools into the electronic medical records is a complex process that requires thorough quality assurance. Whether the proposed opportunistic screening approach improves effectiveness and outcome of lung cancer screening eligible individuals needs to be evaluated in clinical trials.

In conclusion, deep learning allows for fully automated, opportunistic SLD screening from lung cancer screening chest CTs, which might be of particular importance to identify lean individuals with SLD but without traditional risk factors who may otherwise go unnoticed. Extracting this readily available but currently unused information may help to guide preventive strategies and initiate further workup to reduce morbidity and mortality.

## Author contributions

**Jakob Weiss, Simon Bernatz, Michael T. Lu, Hugo J. W. L. Aerts**: Conceptualization. **Jakob Weiss, Simon Bernatz, Justin Johnson, Vamsi Thiriveedhi, Andriy Fedorov**: Data curation. **Jakob Weiss, Simon Bernatz**: Formal analysis. **Michael T. Lu, Hugo J. W. L. Aerts**: Funding acquisition. **Jakob Weiss, Simon Bernatz**: Investigation. **Jakob Weiss, Michael T. Lu**: Methodology. **Jakob Weiss**: Project administration. **Raymond H. Mak, Michael T. Lu, Hugo J. W. L. Aerts**: Resources. **Justin Johnson, Andriy Fedorov**: Software. **Michael T. Lu, Hugo J. W. L. Aerts**: Supervision. **Raymond H. Mak, Michael T. Lu, Hugo J. W. L. Aerts**: Validation. **Jakob Weiss, Simon Bernatz**: Visualization. **Jakob Weiss, Simon Bernatz, Michael T. Lu, Hugo J. W. L. Aerts**: Writing—original draft. All authors: Writing—review and edition.

## Conflict of interest statement

The authors declare no conflicts of interest.

## Supporting information




**Figure S1**: All‐cause mortality by steatotic liver disease
**Figure S2**: Change in hepatic fat fraction between baseline and 1‐year follow‐up scan
**Figure S3**: Association between steatotic liver disease and mortality at baseline for women
**Figure S4**: Association between steatotic liver disease and mortality at baseline for men
**Figure S5**: Scatter plot for continues BMI vs. hepatic fat fraction

## Data Availability

All NLST CT images, TotalSegmentator segmentations of those images, and the per‐segment radiomics features are publicly available from NCI Imaging Data Commons https://portal.imaging.datacommons.cancer.gov/. Access instructions, as well as radiomics features for all segmented structures extracted into CSV format are available from Zenodo [23].
