# Supplementary material for: Opportunistic assessment of steatotic liver disease in lung cancer screening eligible individuals
Source: J Intern Med. 2025 Jan 27;297(3):276–88. doi: 10.1111/joim.20053 (PMC11846076; doi:10.1111/joim.20053)
Supplement: Supplementary file 1 — Figure S1: All‐cause mortality by steatotic liver disease Figure S2: Change in hepatic fat fraction between baseline and 1‐year follow‐up scan Figure S3: Association between steatotic liver disease and mortality at baseline for women Figure S4: Association between steatotic liver disease and mortality at baseline for men Figure S5: Scatter plot for continues BMI vs. hepatic fat fraction [file JOIM-297-276-s001.docx]

**Opportunistic Assessment of Steatotic Liver Disease in Lung Cancer Screening Eligible Individuals**

Jakob Weiss, Simon Bernatz, Justin Johnson, Vamsi Thiriveedhi, Raymond H. Mak, Andriy Fedorov, Michael T. Lu, Hugo J.W.L. Aerts

**SUPPLEMENTAL METHODS**

- **Supplemental Methods:** CT image acquisition

**SUPPLEMENTAL FIGURES**

- **Supplemental Figure 1:** All-cause mortality by steatotic liver disease
- **Supplemental Figure 2:** Change in hepatic fat fraction between baseline and 1-year follow-up scan
- **Supplemental Figure 3:** Association between steatotic liver disease and mortality at baseline for women
- **Supplemental Figure 4:** Association between steatotic liver disease and mortality at baseline for men
- **Supplemental Figure 5:** Scatter plot for continues BMI vs. hepatic fat fraction

**SUPPLEMENTAL REFERENCES**

**Supplemental methods**

**CT image acquisition**

To ensure a comparable imaging standard across all NLST study sites, all scanners (GE Medical Systems, Philips, Siemens, Toshiba) and imaging protocols were certified for use in the trial and protocol settings complied with the American College of Radiology guidelines as previously published in detail[^1^](https://paperpile.com/c/1IhVLf/4uKg). In brief, as per study protocol images were acquired in supine position with arms elevated above the head and in suspended maximum inspiration. Images were acquired with a tube voltage of 120-140 kVp, a tube current-time product of 40-80 mAs and a collimation ≤2.5 mm. From the acquired data axial series were reconstrued with a nominal reconstructed section with between 1.0-3.2 mm using a soft tissue or hard kernels.

**Supplemental Figures**

**All-cause mortality by steatotic liver disease**


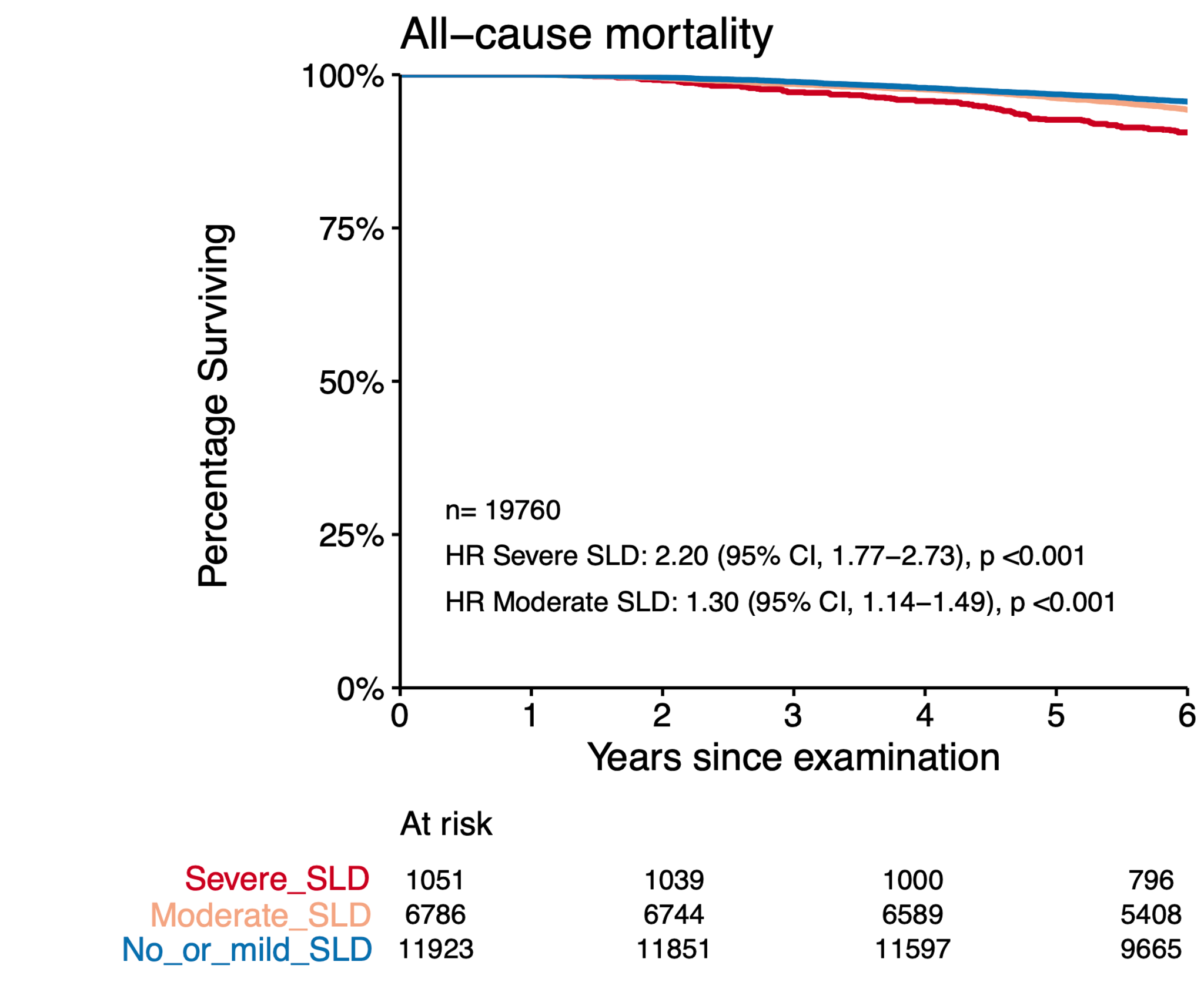


**Supplemental Figure 1:** Kaplan-Meier curve and HR for different SLD fat fraction groups (severe: fat fraction >20%; moderate: fat fraction 6-20%, no or mild [reference]: fat fraction ≤5%), which indicate a graded association between increasing hepatic fat fraction and all-cause mortality.

SLD: steatotic liver disease; HR: hazard ratio

**Change in hepatic fat fraction between baseline and 1-year follow-up scan**


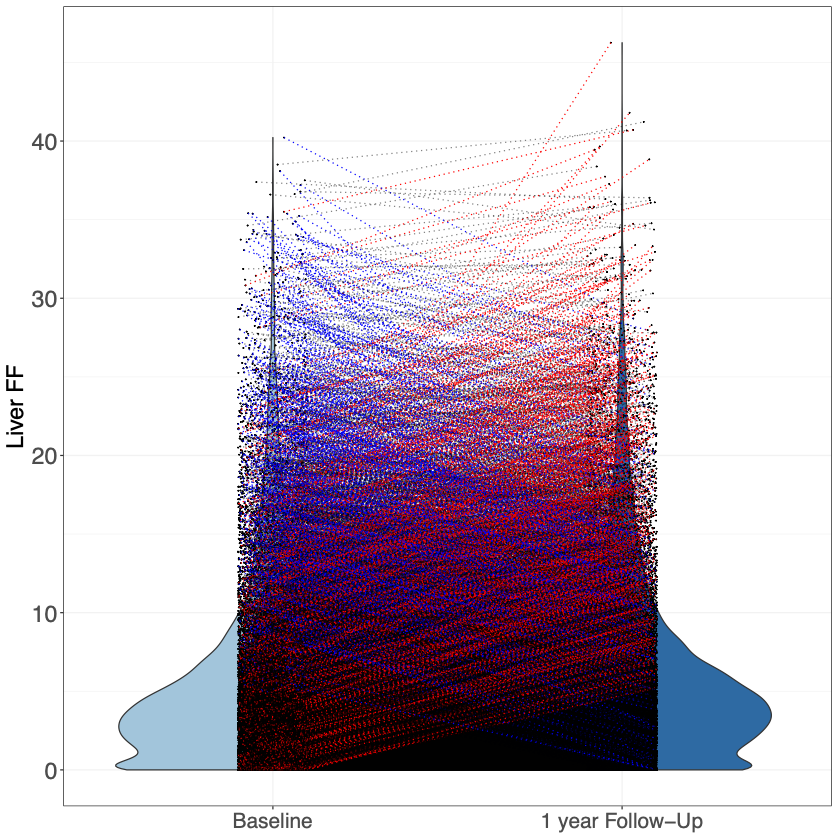


**Supplemental Figure 2:** Individuals who gained at least 5% absolute fat fraction are depicted in red, those who lost at least 5% absolute fat fraction are depicted in blue, and stable patients are depicted in black.

FF=fat fraction

**Association between steatotic liver disease and mortality at baseline for women**


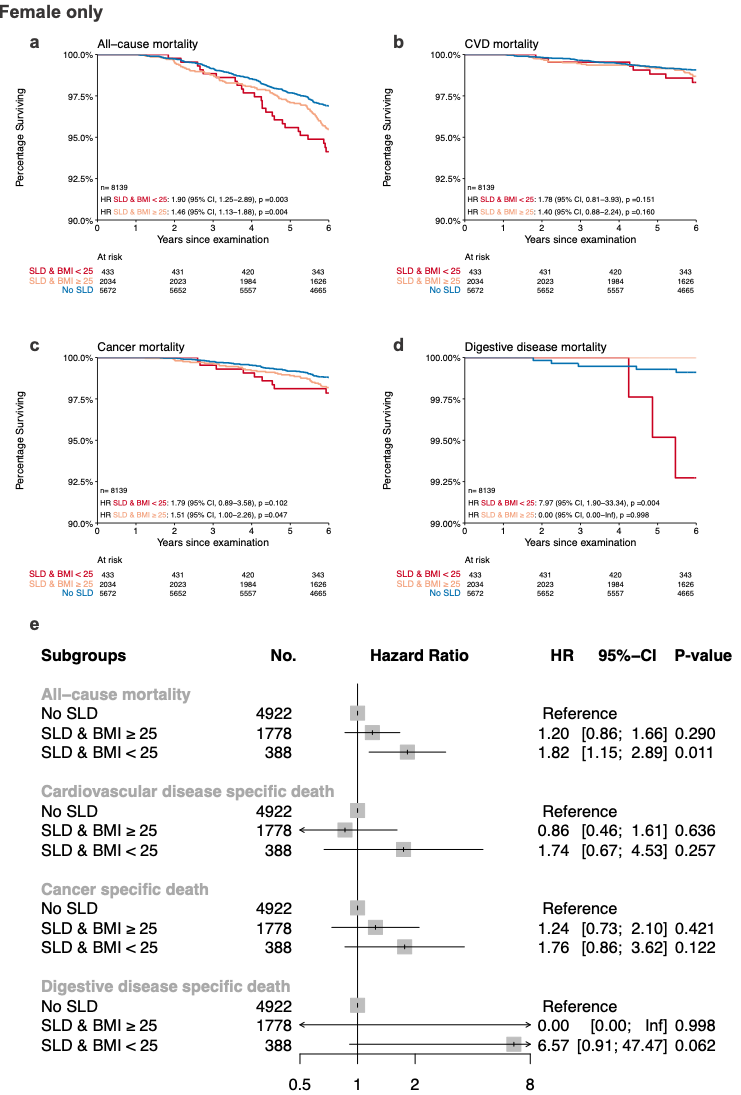


**Supplemental Figure 3:** Kaplan Meier survival curves for steatotic liver disease by body mass index (BMI) groups for **a**) all-cause, **b**) cardiovascular disease (CVD), **c**) cancer, and **d)** digestive disease mortality in women only. The inserts show univariate Cox regression analyses versus the reference of no steatotic liver disease. **e**) Forest plots with multivariable-adjusted hazard ratios (HR) and 95% confidence intervals (CI) including age, BMI, sex, race, smoking status, pack-years, prevalent hypertension, prevalent type II diabetes, past myocardial infarction, stroke, and alcohol consumption as covariates.

BMI, Body Mass Index; CVD, Cardiovascular Disease; HR, Hazard Ratio; SLD, Steatotic Liver Disease.

**Association between steatotic liver disease and mortality at baseline for men**


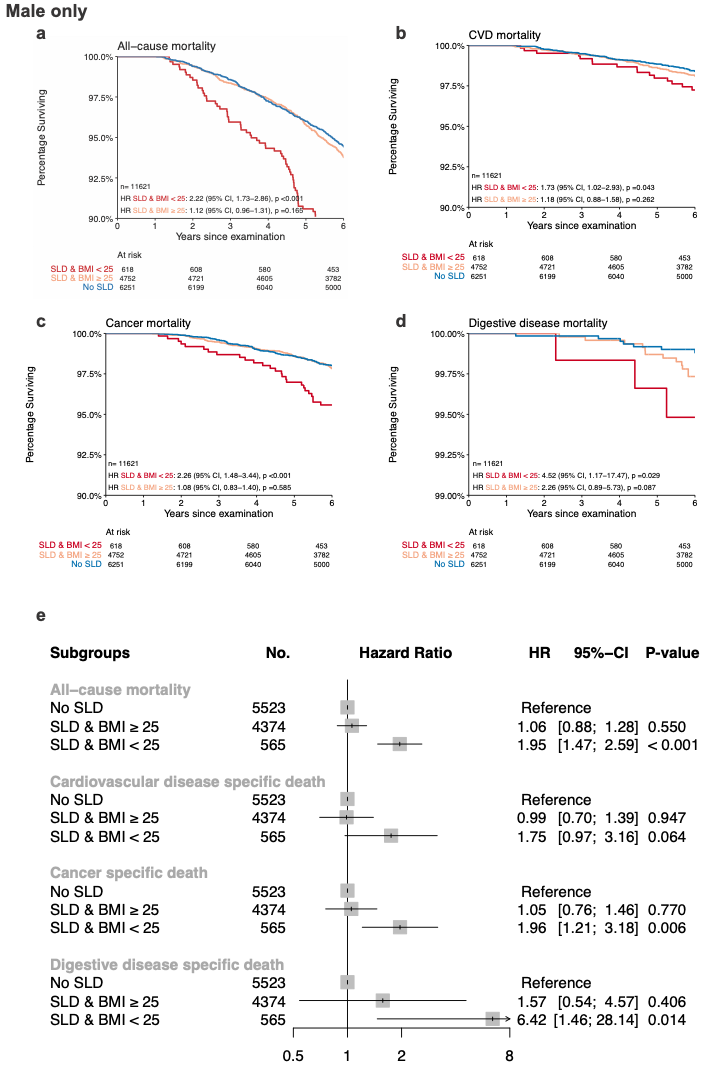


**Supplemental Figure 4:** Kaplan Meier survival curves for steatotic liver disease by body mass index (BMI) groups for **a**) all-cause, **b**) cardiovascular disease (CVD), **c**) cancer, and **d)** digestive disease mortality in men only. The inserts show univariate Cox regression analyses versus the reference of no steatotic liver disease. **e**) Forest plots with multivariable-adjusted hazard ratios (HR) and 95% confidence intervals (CI) including age, BMI, sex, race, smoking status, pack-years, prevalent hypertension, prevalent type II diabetes, past myocardial infarction, stroke, and alcohol consumption as covariates.

BMI, Body Mass Index; CVD, Cardiovascular Disease; HR, Hazard Ratio; SLD, Steatotic Liver Disease.

**Scatter plot for continues BMI vs. hepatic fat fraction**


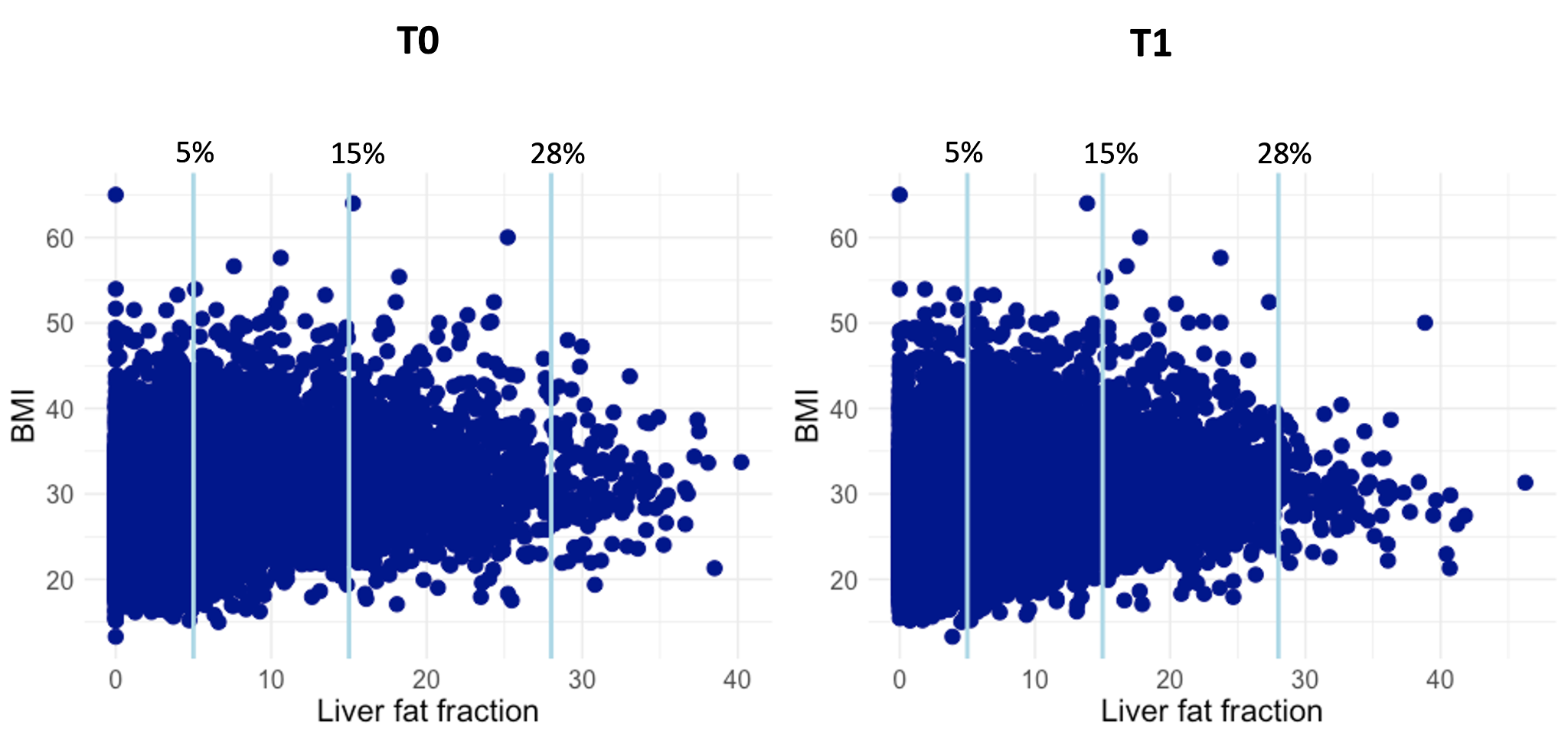


**Supplemental Figure 5:** Scatter plot for continuous BMI vs. hepatic fat fraction for T0 and T1. Vertical lines in light blue indicate fat fraction thresholds for mild (5%), moderate (15%) and severe (28%) steatotic liver disease.

BMI: Body mass index

**Supplemental references**

1. [The National Lung Screening Trial: Overview and Study Design1. *Radiology* **258**, 243 (2011).](http://paperpile.com/b/1IhVLf/4uKg)
